# Contraceptive practices among married women of reproductive age in Bangladesh: a review of the evidence

**DOI:** 10.1186/s12978-017-0333-2

**Published:** 2017-06-06

**Authors:** Fauzia Akhter Huda, Yolande Robertson, Sabiha Chowdhuri, Bidhan Krishna Sarker, Laura Reichenbach, Ratana Somrongthong

**Affiliations:** 1 Maternal and Child Health Division, icddr,b, Dhaka, Bangladesh; 2 Melbourne Academic Centre for Health, University of Melbourne, Melbourne, Australia; 3 The Sidney Kimmel Comprehensive Cancer Center, School of Medicine, Johns Hopkins University, Baltimore, MD USA; 4 Population Council, Washington, DC USA; 5 College of Public Health Sciences, Chulalongkorn University, Room 1012, Floor 10, Institute Building 3, Bangkok, Thailand

**Keywords:** Family planning, Contraceptive practices, Married women of reproductive age, Bangladesh

## Abstract

**Background:**

Bangladesh has experienced a sevenfold increase in its contraceptive prevalence rate (CPR) in less than forty years from 8% in 1975 to 62% in 2014. However, despite this progress, almost one-third of pregnancies are still unintended which may be attributed to unmet need for family planning and discontinuation and switching of methods after initiation of their use.

**Methods:**

We conducted an extensive literature review on contraceptive use among married women of reproductive age (MWRA) in Bangladesh. A total of 263 articles were identified through database search and after final screening ten articles were included in this synthesis.

**Results:**

Findings showed that method discontinuation and switching, method failure, and method mix may offset achievements in the CPR. Most of the women know of at least one contraceptive method. Oral pill is the most widely used (27%) method, followed by injectables (12.4%), condoms (6.4%), female sterilization (4.6%), male sterilization (1.2%), implants (1.7%), and IUDs (0.6%). There has been a decline in the use of long acting and permanent methods over the last two decades. Within 12 months of initiation, the rate of method discontinuation particularly the short-acting methods remain high at 36%. It is important to recognize the trends as married Bangladeshi women, on average, wanted 1.6 children, but the rate of actual children was 2.3.

**Conclusions:**

A renewed commitment from government bodies and independent organizations is needed to implement and monitor family planning strategies in order to ensure the adherence to and provision of the most appropriate contraceptive method for couples.

## Plain English summary

Bangladesh has experienced a sevenfold increase in its contraceptive prevalence rate (CPR) in less than forty years from 8% in 1975 to 62% in 2014. However, despite this progress, almost one-third of pregnancies are still unintended which may be attributed to unmet need for family planning and discontinuation and switching of methods.

We conducted an extensive literature review on contraceptive use among married women of reproductive age (MWRA) in Bangladesh. A total of 263 articles were identified through database search and after final screening ten articles were included in this synthesis.

Findings showed that method discontinuation and switching, method failure, and method mix may offset achievements in the CPR. Most of the women know of at least one contraceptive method. Oral pill is the most widely used (27%) method, followed by injectables (12.4%), condoms (6.4%), female sterilization (4.6%), male sterilization (1.2%), implants (1.7%), and IUDs (0.6%). There has been a decline in the use of long acting and permanent methods over the last two decades. Within 12 months of initiation, the rate of method discontinuation particularly the short-acting methods remain high at 36%. On average, married Bangladeshi women wanted 1.6 children, but the rate of actual children was 2.3.

In conclusion, a renewed commitment from government bodies and independent organizations is needed to implement and monitor family planning strategies in order to ensure the adherence to and provision of the most appropriate contraceptive method for couples.

## Background

In Bangladesh, an estimated three in five married women currently use a method of contraception. The country experienced an impressive sevenfold increase in its contraceptive prevalence rate (CPR) in less than forty years, from 8% in 1975 to 62% in 2014 [1, 2]. The CPR plays a significant role in assessing the demographic impact of family planning (FP) programs [3]. However, it is imperative to recognize that fertility is not solely dependent on the prevalence of contraceptive use but also on contraceptive use-effectiveness and user adherence [3]. Married women in the country are having 0.7 more children than they desire, meaning that the total fertility rate (TFR) would be 30% lower if unplanned pregnancies were avoided [3]. While this may also be explained by unmet need for family planning, which is equally important to explore the effectiveness of family planning programs in addressing issues related to contraceptive method use. These issues include method discontinuation and switching, method mix, and method failure [3]. Despite Bangladesh’s impressive gains in CPR, it is important to understand why, almost one-third of pregnancies are still unintended.

Ideally, family planning programs should offer a wide range of methods and appropriate counseling, so that users can make an informed choice and easy access to quality follow-up services since these factors are associated with method satisfaction, continuation and switching [4]. Studies of contraceptive use dynamics typically address the mentioned three aspects in order to provide guidance for improving services. Evidence from these studies has a number of programmatic implications, including better monitoring and evaluation of program activities, improved effectiveness in meeting the needs of users, and more generally, improved ability of governments to achieve goals set for total fertility, and for maternal and child health services [4].

Although numerous studies on discontinuation and switching and fertility intention in Bangladesh exist, these studies have not been systematically collated and reviewed so as to determine the factors associated with women’s contraceptive practices and fertility behaviors. The aim of this research was to develop a country profile report that provides a comprehensive view of the current state of contraceptive practices among married women in Bangladesh. The overall goal was to identify, generate, communicate and use a robust body of research-based evidence for the development of more effective policy and program for family planning services in Bangladesh.

## Review method

### Search strategy

A literature search was performed using the databases PubMed/MEDLINE, HINARI, JSTOR and Google Scholar. We searched for articles that reported on contraceptive use in Bangladesh only, by including the following keywords: married women of reproductive age (MWRA) between 15 and 49 years; unintended pregnancy; contraceptive use; method mix; demand and practices for family planning; discontinuation and switching; fertility preferences and intentions. Articles in English were accepted.

We also retrieved and reviewed published and unpublished documents and literature on contraceptive behavior and unmet need for family planning in Bangladesh. This was done by identifying local research-based organizations working on family planning and collecting documents such as posters, flipcharts, books and leaflets.

National level survey reports such as the Bangladesh Demographic and Health Survey (BDHS) 2014, BDHS 2011, BDHS 2007, and the Bangladesh Maternal Mortality Survey (BMMS) 2010 [1, 2, 5, 6] were also included. We also reviewed the Government Health, Population, and Nutrition Sector Development Program (HPNSDP) 2011–2016 [7] document to identify planned activities that addressed family planning and contraceptive use, and specifically, those that addressed discontinuation and switching of contraceptive methods.

### Criteria for inclusion

We included articles in the review if they had subheading topics from the standard outline provided by STEP UP (Strengthening Evidence for Programming on Unintended Pregnancy) that were adapted to Bangladesh (http://stepup.popcouncil.org). We also included studies, publications and documents on family planning focusing on married women of reproductive age (15–49 years) in Bangladesh from the year 2000 onward and were written in or translated into English.

### Criteria for exclusion

We excluded articles if they focused on the following: a) access to and quality of family planning services and information; b) costs to clients to access family planning services; c) availability, quality, and costs of family planning services by sector; d) financing and delivery mechanisms of family planning services; e) information and communications technology (ICT), E-health/M-health and media for information, education and communication (IEC) and social behavior change and communication (SBCC) on family planning; and f) family planning commodities procurement and logistics supply by sector.

### Data analysis

Variation in study methodologies prevented further quantitative analysis; instead, a narrative review of the evidence is presented in this article.

## Results

**Fig. 1:**
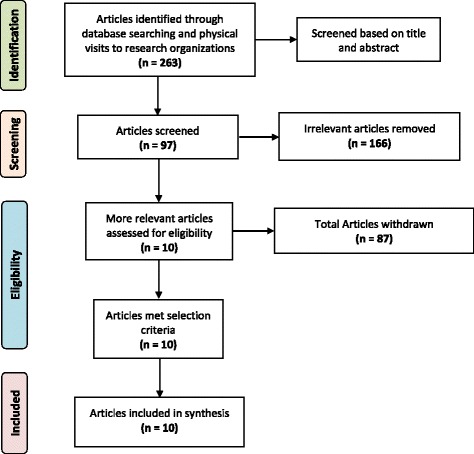
PRISMA flow diagram of study selection process

Of the 263 articles retrieved in our search, 97 were retained to screen full text and 10 articles were included for the review (Fig. 1). Descriptive information of the included articles is presented in Table 1. Seven papers used national level data from the BDHS for analysis, and three collected data from primarily rural settings in Bangladesh.

**Table 1:** Description of the studies reviewed

Reference	Study Type	Sample Size	Study objectives
BDHS 2014	Seventh national level survey based on a two-stage stratified sample of households	18,000 ever married women age 15–49 years	To provide up to date information on fertility and childhood mortality levels; fertility preferences; awareness, approval, and use of family planning methods; maternal and child health including breastfeeding practices, nutrition levels, and newborn care; knowledge and attitudes towards HIV/AIDS and STIs; and community level data on accessibility and availability of health and family planning services.
BDHS 2011	Nationally representative two-stage stratified sample of households	17,842 women age 12–49 years	To provide up to date information on fertility, childhood mortality, fertility preferences, awareness, approval, use of FP methods, maternal and child health, knowledge and attitudes towards HIV/AIDS and STIs, accessibility and availability of health and FP services.
BDHS 2007	Nationally representative survey	10,996 women aged 15–49 and 3,771 men aged 15–54 years	To provide up-to-date information on fertility and childhood mortality levels; fertility preferences; awareness, approval, and use of family planning methods; breastfeeding practices; nutrition levels; maternal and child health; awareness of HIV/AIDS and other sexually transmitted diseases; knowledge of tuberculosis; and domestic violence.
Ferdousi 2010	Descriptive cross- sectional study	272 rural women aged 15–49 years	To explore the proportion of unmet need of family planning among the 272 married woman of reproductive age living in a rural area. The study also explored the contraceptive prevalence rate, reasons for not using contraceptives, and the methods of contraceptives used by the women in the study.
Streatfield 2012	Secondary analysis of Matlab DSS and RKS longitudinal data	2,709 women aged 13–49 years	To examine method use behaviour of 2,709 women, who were in the study almost over their entire reproductive years. They also took a cutoff point (2008) and examined the method use pattern of various age groups in that year.
Kamal 2011	Cross- sectional study, analysis of BDHS 2007 data	3, 866 rural women aged 15–49 years	To determine the prevalence of unintended pregnancy and socioeconomic correlates such as pregnancy order, age, religion, ever use of FP methods, region and wealth index to determine if they were associated with unintended pregnancy among rural women
Saha 2007	BDHS 1999–2000 data, and Matlab DSS area data collected between 1978 and 2001 were analyzed.	BDHS 1999–2000: 10,544 ever-married women aged 10–49, of whom 9,696 were currently married and aged 15–49 years). Matlab DSS (1978 and 2001) (dealt with over 200,000 population). Women of aged 15–49 years.	To explain the lack of change in fertility in Bangladesh despite the increase in contraceptive prevalence rate and to examine relationships among contraceptive prevalence, the abortion ratio, desired fertility and total fertility by secondary analysis of data. They determined the factors that can be attributed to explain the difference between the national TFR and desired fertility.
Reza 2001	Thesis paper, analysis of BDHS 1996–97 data	3,312 currently married men aged 15–59 years	To determine the factors influencing men’s fertility preference in Bangladesh. They examined factors such as region of residence, education, age, occupation, gender preference, religion, influence of mass media and interspousal communication to show their association with fertility preference of men.
Islam 2010	Secondary data analysis on three consecutive Bangladesh Demographic and Health Surveys (BDHSs) (1996–1997, 1999–2000 and 2004)	Sample drawn from integrated Multipurpose Master Sample (IMPS) created by Bangladesh Bureau of Statistics (BBS).	To assess how fertility patterns in two high fertility regions (Sylhet and Chittagong) differ from rest of the country through a marriage cohort analysis and examined the factors determining the higher parity in these two regions. Among other factors, they also examined the effect of son preference on fertility in these regions
Gipson 2009	Analysis of cross-sectional and longitudinal utilized surveillance data from the Sample Registration System (SRS) of icddr,b. From two thanas of Jessore District Bangladesh.	3,052 rural Bangladeshi couples (women of reproductive age 15–49 and their husbands- age not specified)	To investigate the influence of husbands’ and wives’ fertility preferences on the likelihood of a subsequent pregnancy in the period 1998–2003.

### Awareness of family planning

Results from the 2014 BDHS showed that knowledge of family planning methods were widespread throughout Bangladesh, with almost all ever-married and currently married women were able to identify at least one modern method of contraception [1]. Seven out of ten women could mention at least one traditional method and, on an average they were able to name 7.4 different methods of contraception [5].

### Use of family planning methods

**Fig. 2:**
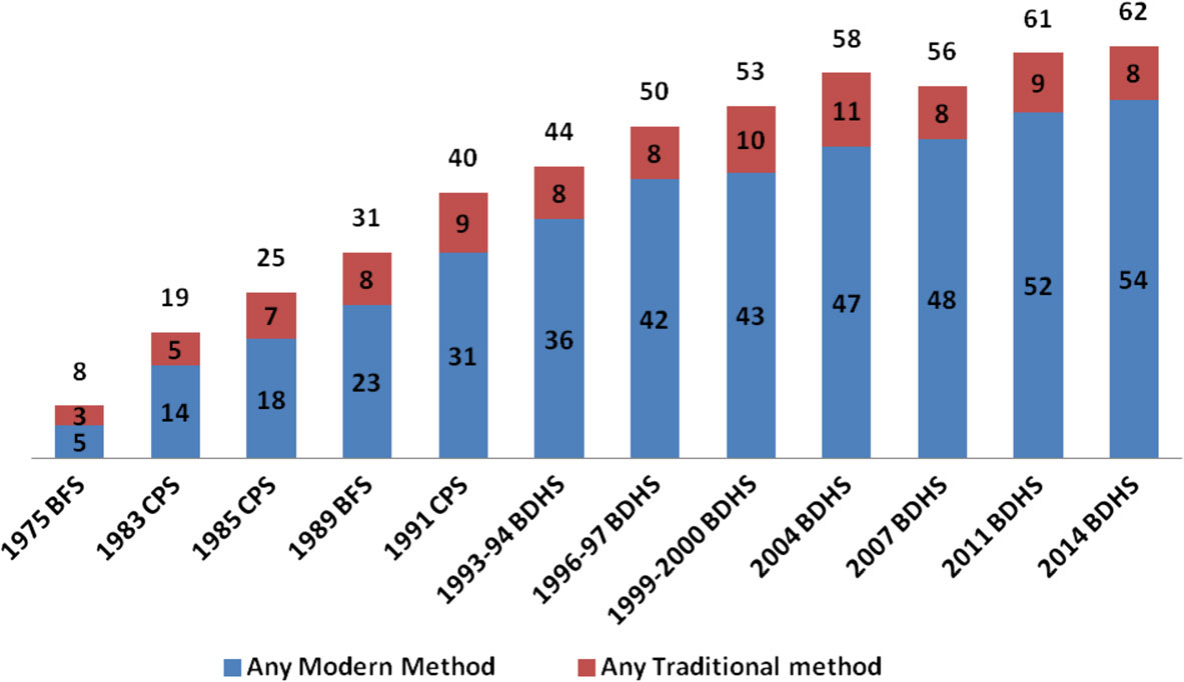
Percent in contraceptive use among currently married women in Bangladesh, 1975–2014 (BDHS 2014)

The most recent BDHS reports that 62% of currently married women aged 15–49 years in Bangladesh are using any contraceptive methods, with 54% using a modern method [1]. The rate of contraceptive use has increased significantly from eight percent in 1975, to 62% in 2014. However, this rate has slowed since 2004, increasing by four percentages over the past decade; while the use of modern methods has increased by seven percentages from 47 to 54% in the same period [1, 2, 5] (Fig. 2).

Current use of FP methods among women varies by place of residence, age and parity. Use of any modern contraceptive method is higher in urban than rural areas, with 66 and 61% reported use, respectively [1]. For age, a bell shaped pattern was observed with 47% of currently married women between 15–19 years of age to a peak usage of 65% at age 30–34 and then a steady decrease to 25% at age 45–49 [1]. Any modern contraceptive method use was also found related to women’s parity; increasing parity up to four living children was associated with an increase in use of contraceptive methods [1].

Ferdousi et al. (2010) identified fear of side effects as a major reason for not using contraceptives (46%) followed by religious reasons (12%) and husbands or family disapproval (11%) [8].

### Method mix

The pill remains the most widely used method (27%), followed by injectables (12%), condoms (6%), and female sterilization (5%) [1]. Only eight percent of currently married couples use long-acting reversible contraceptives (LARC) such as, implants or intrauterine devices (IUDs), or a permanent method of contraception such as female or male sterilization. Use of long acting and permanent methods peaked at 30% in 1991 but declined steadily and stabilized at the current rate of 8% in 2007 [1, 2, 5]. While there has been a slight increase in the use of male sterilization since 2004, which is safer and cheaper than female sterilization, the usage rates still remain very low (1%) [1]. Knowledge on and use of emergency contraceptive pill (ECP) among the currently married women was found lower; fourteen percent have ever heard of it, 13% of them have ever used it, and 6% used it within the last 12 months. [1].

### Discontinuation and Switching

**Fig. 3:**
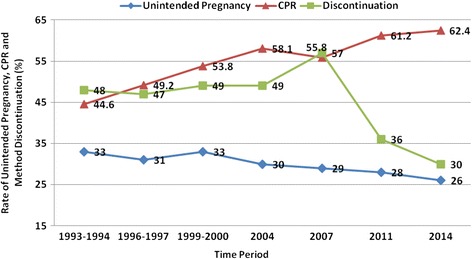
Trends of unintended pregnancy, contraceptive prevalence rate and method discontinuation in Bangladesh from 1993 to 2014 (BDHS 2014)

Contraceptive users who discontinue their method use within one year declined consistently from 1993 and peaked to 57% in 2007, and dropped to 30% in 2014, which is the lowest recorded rate in the BDHS (Fig. 3). This rate is higher among users of short acting methods such as condoms (40%), and the pill (34%) than for longer-term methods such as implants (7%). All method discontinuation rate declined from 36% in 2011 to the current rate of 30% in 2014 [1]. Reasons for discontinuation included desire to become pregnant (31%), followed by side effects (26%) and accidental pregnancies (14%). However, the reasons for discontinuation varied depending on the method of contraception (Table 2).

**Table 2:** Reasons for discontinuation of contraceptive method use among married women of reproductive age in Bangladesh (BDHS 2014)

Reason	Pill	IUD	Injectable	Implant	Male Condom	Periodic Abstinence	Withdrawal/Coitus Interrupt	All Methods
Wanted to become pregnant	33.8	19.4	23.8	20.9	34	26.2	26.9	31.1
Side effects	24.5	46.7	45.5	51.1	11.1	1.2	1.6	25.8
Became Pregnant while using method	16.5	0	5.6	1.1	12.8	19.1	17.8	13.8
Infrequent Sex/husband away	9.4	0	5.1	0.4	7.8	4.9	7.1	7.8
Wanted a more effective method	3.5	4.3	2.7	6.9	6.7	17.9	11.3	4.8
Inconvenient to use	3.7	4.2	2.4	5	11	5.2	15	4.6
Difficult to get pregnant/Menopausal	2.4	2.8	4.8	0	1.3	14.1	4.8	3.4
Husband disapproved	0.6	0	0.5	1.4	9	1.9	10.7	1.9
Marital dissolution/separation	0.8	10.3	0.9	4.6	1.2	1.3	0.5	1
Lack of access/too far	0.4	1.4	2.8	0.3	0.2	0	0	0.8
Cost too much	0.1	0	0.2	0	0.1	0	0	0.1
Up to God/fatalistic	0.1	0	0	0	0	0	0	0.1
Other	0.6	9.6	1.2	8.4	0.3	0.8	0.2	0.9
Don’t know	0	0	0	0	0	0	0	0
Missing	3.6	1.3	4.3	0	3.7	7.4	4	4
Total	100.00	100.00	100.00	100.00	100.00	100.00	100.00	100.00
Number of discontinuation	5,766	73	1,860	157	1,109	566	179	9,731

Source: Adapted from Bangladesh Demographic and Health Survey 2014

Note: Total includes 11 women who used other methods

A study in Matlab, in Chandpur District, showed that on average, women use 2.5 methods over their reproductive lifetime, with a mean number of switches at 13.5 episodes [9].

### Desired fertility

The current preferred family size for women in Bangladesh is 2.2 children, which has declined slightly over the last decade from 2.4 children. The BDHS 2014 reports that 74% of pregnancies in the five years preceding the survey were wanted or intended, and 26% of pregnancies were unintended (15% were mistimed and 11% were unwanted) (Fig. 3). Findings from the multi-variate analysis of one study showed that pregnancy order, age, religion, ever use of contraceptive methods, region and wealth index were significantly associated with pregnancy intention status [10].

In 2014, though the total wanted fertility rate in the country was 1.7 children, while the actual fertility rate was found 2.3 [1]. Findings from one study showed that the gap in fertility desire is mostly due to preference for male children, security against infant and child mortality, lack of quality family planning services, and ineffective use of methods [11]. It was also found that with each parity level, fertility was highest for women without sons, and decreased with the increase in number of sons [11].

One study reported a dependency on sons during old age, as the sons are economically more productive than daughters. Women with a higher proportion of sons were less likely to want more children and the contraceptive method use was more likely among them [12, 13]. Sons can also bring a dowry to the family through marriage, although the practice is technically illegal in Bangladesh [13]. The number of sons in a family also has a significant effect on the timing of a third birth in many parts of the country. One study found that the event of a third birth was later for women who already had one or two sons. Data also showed that early marriage, large age difference between spouses, early childbearing, and reduced educational and employment opportunities for women reinforce women’s dependency on sons, though these practices are gradually decreasing [14].

### Unmet need for family planning

Unmet need for family planning refers to fecund women who are not using contraception but who wish to postpone the next birth (spacing) or stop childbearing altogether (limiting). Unmet need has decreased from 14% in 2011 to 12% in 2014, of which five percent was for spacing and seven percent was for limiting births [1].

Unmet need varies with increasing age and area of residence. Women aged 15–19 had 10% more unmet need than women aged 45–49 (17 and 7%, respectively). Similarly, rural women had a higher unmet need than urban women (13 and 10%, respectively) [1].

## Discussion

This review has showed that majority of women are aware of several family planning methods but they often do not know about the correct use of methods and have misconceptions or fears about side effects. Although the contraceptive prevalence rate has increased sevenfold across four decades, it is offset by discontinuation, switching, and incorrect use. The method mix is predominated by short-term methods, with a consistent decline in the use of long acting and permanent methods. Recognizing that this has a significant impact on long term health outcomes for women, the government has already taken elaborate measures in the new HPNSDP (2011–2016) to promote the use of long acting and permanent methods [7].

Unintended pregnancy has declined gradually, however still comprise about one third of total pregnancies, and the unmet need for family planning lies at 12% [1]. Together, these factors are responsible for the gap between the number of children couples want, and the number that they actually have. Preference for male children and parental concern over infant and child mortality may partially explain the difference between desired family size and fertility [11]. The gap between wanted and actual fertility also represents a woman’s inability to achieve her own reproductive goals. This suggests that currently women in Bangladesh are having 0.7 more children than intended, and the total fertility rate would be 30% lower if all unwanted births were avoided.

Short-term methods such as oral pills, injectables, and condoms were shown to predominate in the method mix, which are not as successful as long acting and permanent methods. Women who are using such methods, and who have already reached their desired family size in their late twenties, are putting themselves at risk of unintended pregnancies for the remaining part of their reproductive lives. Higher use of short-term methods and female sterilization, compared to lower rate of use of condoms and male sterilization implies a stronger emphasis on female responsibility for birth control.

Even though there have been positive gains in the decreasing proportion of unintended pregnancies (from 33% in 1993 to 26% in 2014), and an increase in CPR by 17% in the same period [1, 2], there is still a significant number of women who are not covered in this progress.

The Government of Bangladesh’s pledge at the London Summit on Family Planning includes increasing contraceptive access and use among poor people in urban and rural areas, improving choice and availability of long acting and permanent methods (LAPMs) [15]. The Government plans to increase LAPM use through an increase in supply of materials and instruments needed for LAPMs, targeting low performing areas, increasing awareness and outsourcing programs to non-governmental organizations (NGOs) to cover hard to reach and low performing areas [7].

Another factor that contributes to the rate of unintended pregnancies is the high level of discontinuation across all methods, with 30% of users terminating use after one year [1]. It is encouraging that the discontinuation rate has decreased by 27% since 2007; however the fact that one in three women discontinue use of their chosen contraceptive method after a year is still significant. This could be addressed through more marketing to promote newer progestin based contraceptives with nominal side effects that are known to be effective, yet have low usage rates [16]. A study in Uganda showed that two thirds of the respondents (66%) felt that receiving messages on mobile phones helped them with contraceptive adherence or continuation decisions; in managing side effects (52%); and in using the method more effectively (66%) [17].

### Limitations

This review had two main limitations. Firstly, the studies reviewed were cross sectional in design and were not able to draw causal references. Furthermore, many of the studies reported on data from 2010 or earlier, which may not give an accurate view of the situation in Bangladesh in 2017.

## Conclusion

Although success of the family planning program in Bangladesh has been widely acclaimed, many challenges still remain. Several demand- and supply-side strategies can help the national family planning program to overcome these challenges. At the same time, a renewed commitment from government bodies to implement and monitor such strategies, as well as to maintain ongoing collaboration with independent organizations is needed. The notable progress in country’s family planning program must be continued and strengthened to reach its goal of replacement level fertility.

### Recommendations

The above findings lead us to some recommendations. Firstly, in order to prevent the gains made in the CPR over the past four decades from being offset by discontinuation and switching of methods, and to promote the use of mix methods, relevant health care providers need to be trained on counseling the couples on method use, options, and accurate information of its risks to allay health concerns. Husbands should be counseled with their wives on long acting and permanent method uptake, and birth planning as they play a major role in decisions to limit/space childbirth and overall issues related to family planning [12]. The importance of counseling also needs to be emphasized in training courses for the health care providers. Additionally, many of the training materials for community level and primary level health care providers, which have not been updated in the past 20 to 30 years [18], need to be updated following the WHO and other international standards and guidelines.

Secondly, in order to sustain the declining trend of method discontinuation, family planning programs should consider restoring field workers’ provision of door to door visit to ensure supplies of contraceptive methods as it has been shown to facilitate continuation of methods [19]. Field workers need to be uniquely placed to provide timely and relevant information to each woman they visit, and to promote the use of government health facilities and family planning services that are available.

Furthermore, introducing newer progestin based contraceptives that are not only more effective but also have fewer side effects, can help reduce discontinuation and thus should be provided as an alternative to users who discontinue method use as a result of side effects [17].

Additionally, well-articulated mobile messages explaining proper use of methods, side effects, discontinuation, method mix, and comprehensive information on long acting and permanent methods, can be developed in collaboration with Directorate General of Family Planning (DGFP), ICT and NGOs, and be disseminated to eligible couples.

Finally, mass media, adult-education, and school curriculum need to be used to motivate people to value children irrespective of their sex, to highlight the benefits of small family size, and the important role of girls in the family as well as in the society. Awareness rising activities to reduce preference for male children needs to be implemented. Inter-spousal communication should be encouraged during family planning counseling of couples to influence men’s desired family size and contraceptive method use.

## Abbreviations

BDHS: Bangladesh Demographic Health Survey
BMMS: Bangladesh Maternal Mortality Survey
CPR: Contraceptive prevalence rate
DGFP: Directorate General of Family Planning
FP: Family planning
HPNSDP: Health Population and Nutrition Sector Development Program
ICT: Information and communications technology
IEC: Information, education and communication
IUD: Intra-uterine device
LAPM: Long-acting and permanent methods
MWRA: Married women of reproductive age
NGO: Non-governmental organization
PRISMA: Preferred reporting items for systematic reviews and meta-analyses
SBCC: Social and behaviour change communication
STEP UP: Strengthening Evidence for Programming on Unintended Pregnnacy
TFR: Total fertility rate
WHO: World Health Organization

## References

[1] Bangladesh Demographic and Health Survey 2014 (2015). National Institute of Population Research and Training (NIPORT).

[2] Bangladesh Demographic and Health Survey 2011 (2013). National Institute of Population Research and Training (NIPORT), Dhaka, Bangladesh.

[3] Bairagi R, Islam M, Barua MK (2000). Contraceptive failure: levels, trends and determinants in Matlab, Bangladesh. J Biol Sci.

[4] Bongaarts J, Sinding S (2011). Family planning as an economic investment. Washington D.C.

[5] Bangladesh Demographic and Health Survey 2007 (2009). National Instuitute of Population Research and Training (NIPORT), Dhaka, Bangladesh.

[6] Bangladesh Maternal Mortality and Health Care Survey 2010 (2012). Dhaka, Bangladesh: National Institute of Population Research and Training (NIPORT), MEASURE Evaluation, and icddr,b.

[7] Health, Population, and Nutrition Sector Development Program (2011–2016), Program Implementation Plan. In: MoH&FW, editor. Dhaka: Government of Bangladesh.

[8] Ferdousi SK, Jabbar MA, Hoque SR, Karim SR, Mahmood AR (2010). Unmet Need of Family Planning Among Rural Women in Bangladesh. J Dhaka Med Coll.

[9] Streatfield P, Kamal N, Taisir R, Hasan M (2012). Discontinuation and Switching Patterns in Matlab, Bangladesh. TRAction Activity Report: Secondary Data Analysis on Contraceptive Use.

[10] Kamal M, Islam A (2011). Prevalence and socioeconomic correlates of unintended pregnancy among women in rural Bangladesh. Salud Publica Mex.

[11] Saha U, Bairagi R (2007). Inconsistencies in the relationship between contraceptive use and fertility in Bangladesh. Int Fam Plan Perspect.

[12] Reza R (2001). Factors influencing fertility preferences of men in Bangladesh.

[13] Islam S, Islam MA, Padmadas SS (2010). High fertility regions in Bangladesh: a marriage cohort analysis. J Biosoc Sci.

[14] Gipson JD, Hindin MJ (2009). The effect of husbands’ and wives’ fertility preferences on the likelihood of a subsequent pregnancy, Bangladesh 1998–2003. Popul Stud (Camb).

[15] London Summit on Family Planning (2012). Summary of Commitments.

[16] Jain AK, Obare F, RamaRao S, Askew I (2013). Reducing Unmet Need by Supporting Women with Met Need. Int Perspect Sex Reprod Health.

[17] Walakira B, Lubale YAM, Balidawa F, Nalule S, Githinji F (2013). Can mobile phone text messaging increase uptake of family planning services in Uganda?.

[18] Streatfield PK, Kamal N (2013). Population and Family Planning in Bangladesh. J Pak Med Assoc.

[19] Hossain MB (2005). Analysing the relationship between family planning workers’ contact and contraceptive switching in rural Bangladesh using multilevel modeling. J Biosoc Sci.

